# Performance and consistency of a fluorescence-based high-throughput screening assay for use in *Babesia* drug screening in mice

**DOI:** 10.1038/s41598-017-13052-5

**Published:** 2017-10-16

**Authors:** Mohamed Abdo Rizk, Shimaa Abd El-Salam El-Sayed, Mahmoud AbouLaila, Rasha Eltaysh, Naoaki Yokoyama, Ikuo Igarashi

**Affiliations:** 1 National Research Center for Protozoan Diseases, Obihiro University of Agriculture and Veterinary Medicine, Inada-Cho, Obihiro, Hokkaido, 080-8555 Japan; 2 Department of Internal Medicine and Infectious Diseases, Faculty of Veterinary Medicine, Mansoura University, Mansoura, 35516 Egypt; 3 Department of Biochemistry and Chemistry of Nutrition, Faculty of Veterinary Medicine, Mansoura University, Mansoura, 35516 Egypt; 4 Department of Parasitology, Faculty of Veterinary Medicine, University of Sadat City, Sadat City, 32511 Minoufiya, Egypt; 5 Department of Pharmacology, Faculty of Veterinary Medicine, Mansoura University, Mansoura, 35516 Egypt

## Abstract

In this study, we evaluated the validity of a fluorescence-based assay using SYBR Green I (SG I) stain for screening antibabesial compounds against *B. microti* in mice. Two different hematocrits (HCTs; 2.5% and 5%) were used. Correlating relative fluorescence units (RFUs) with parasitemia showed significant linear relationships with R^2^ values of 0.97 and 0.99 at HCTs of 2.5% and 5%, respectively. Meanwhile, the Z′ factors in a high-throughput screening (HTS) assay were within the permissible limit (≥0.5) at 2.5% HCT and lower than this value at 5% HCT. Taken together, the highest signal-to-noise (S/N) ratios were obtained at 2.5% HCT; therefore, we concluded that 2.5% was the best HCT for applying fluorescence assay in antibabesial drug screening in mice. Additionally, positive control mice and those treated with diminazene aceturate, pyronaridine tetraphosphate, and an allicin/diminazene aceturate combination showed peak parasitemia and fluorescence values on the same day post-inoculation. Moreover, using different concentrations of SG I revealed that the optimal concentration was 2x. In summary, considering that all experiments were applied under optimal laboratory conditions, fluorescence assay at 2.5% HCT using 2x SG I for *B. microti* parasite offers a novel approach for drug screening in mice.

## Introduction

Babesiosis is a tick-borne disease of great economic importance in the animal industry. The disease is caused by intraerythrocytic protozoan parasites of the genus *Babesia*, which affects animals and humans worldwide^1^. *Babesia bovis* (*B. bovis*) and *Babesia bigemina* (*B. bigemina*) are considered the main causative agents for bovine babesiosis^1^. Moreover, *Theileria equi* (*T. equi*) and *B. caballi* are the causative agents of equine piroplasmosis, which is considered one of the most important protozoan diseases affecting horses, mules, and donkeys^1^. The disease is typified in general by high fever, anorexia, acceleration of the heart and respiratory rates, a marked drop in milk yield, general circulatory shock, hemoglobinuria, pale mucus membranes, and dry yellow feces or bloody stained feces; in severe cases, multi-organ failure might occur, resulting in death^2,3^. The drugs currently used in the field against babesiosis have been shown to have intolerable toxic effects, and resistance against them has been developed^4^. The disease’s severe clinical signs and lack of treatment obligate the researcher to focus on new treatment strategies.

*Babesia microti* (*B. microti*) causes an infection in rodents and discriminated as the most common cause of human babesiosis; it also has served as a useful experimental model for development novel hits for treatment of animal babesiosis^5–7^. Potential novel antibabesial agents are usually evaluated in mice before clinical trials are conducted in the field. At present, microscopic examination of Giemsa-stained thin blood smears from treated and untreated mice is the gold standard for evaluating the growth-inhibiting effects of drug candidates based on the dynamics of parasitemia^8^. However, this method is tedious and time-consuming. Therefore, developing a new, alternate method that provides simplicity, accuracy, and automatic analysis should be a priority.

Recently, our laboratory established a novel *Babesia* fluorescence assay (*B*FA) for large-scale drug screening against *Babesia and Theileria* parasites *in vitro* ^9,10^. The assay depends on using SYBR Green I (SG I) stain, which binds to the double-stranded DNA of parasites^11^. The validation of this assay depends on the statistical parameters of high-throughput screening assay (HTS), including Z′ factor (Z′^),^ signal-to-noise (S/N) ratio, coefficient of variation at the maximum signal (% CV_max_, positive control), and coefficient of variation at the minimum signal (% CV_min_, negative control)^11^. The assay has not yet been evaluated for drug screening against *B. microti* in mice. Therefore, in this study, we evaluated the validation of *B*FA assay for drug screening against the growth of *B. microti* in specific pathogen-free mice. Two antibabesial drugs, including diminazene aceturate (DA) and pyronaridine tetraphosphate (PYR), were used in this study.

## Results

### Determining the optimal HCT for fluorescence assay application in mice

**Figure 1:**
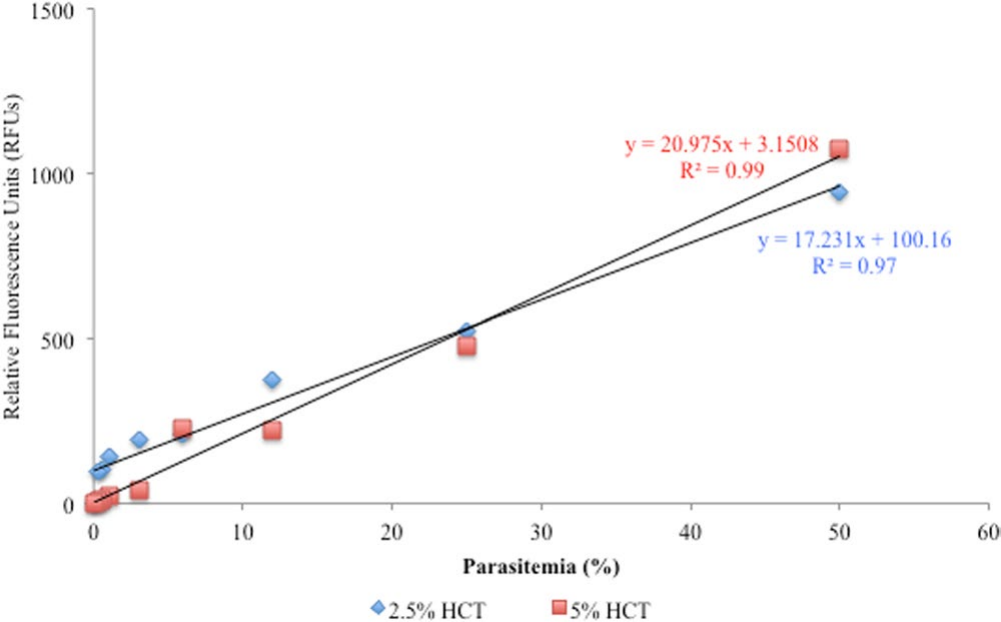
Linearity assessment between relative fluorescence readings and parasitemia percentages of *Babesia microti* pRBCs with different HCTs: 2.5% and 5%. Each value is presented as the mean of triplicates after subtracting the background fluorescence for non-parasitized RBCs from uninfected mice. Gain values are set to 100.

Two different hematocrits (HCTs) (2.5% and 5%) were used to assess the correlation between fluorescence and microscopy values. Significant linear relationships between relative fluorescence units (RFUs) and *B. microti* parasitemia were obtained with both HCTs (Fig. 1). Indeed, both HCTs revealed strong correlations (R^2^ ≥ 0.97) for *B. microti*. Next, high-throughput screening (HTS) assay parameters inclusive of Z′ factor, S/N ratio, % CV_max_, and % CV_min_ were calculated at both HCTs (2.5% and 5%) on days with peak of parasitemia (days 5, 6, and 7) to appraise of the assay quality. The Z′ factors were within the permissible limit (≥0.5) at 2.5% HCT on all examined days. Additionally, 2.5% HCT showed the highest S/N ratios on days with peak parasitemia (Table 1). On the contrary, 5% HCT revealed Z′ factors lower than 0.5 and exhibited the lowest values of S/N ratios in all examined days (Table 1). These results revealed the strength and accuracy of the assay with 2.5% HCT for *B. microti* in mice.

**Table 1:** Statistical parameters for determining the quality of high-throughput screening (HTS) assay in *Babesia microti* parasite with different HCTs.

Parameters	Day 5		Day 6		Day 7	
	HCTs %					
	**2.5**	5	**2.5**	5	**2.5**	5
Z′ factor	**0.89**	−0.16	**0.74**	0.35	**0.84**	0.13
S/N ratio	**122.41**	63.51	**230.97**	117.67	**570.99**	39.56
% CV_max_	**2.53**	33.31	**8.28**	490.57	**4.91**	22.99
% CV_min_	**8.17**	13.74	**36.97**	24.54	**3.01**	15.99

S/N ratio = Signal-to-noise ratio.

% CV_max_ = the coefficient of variation at the maximum signal.

% CV_min_ = the coefficient of variation at the minimum signal.

### Optimal SYBR Green I staining for fluorescence assay

**Figure 2:**
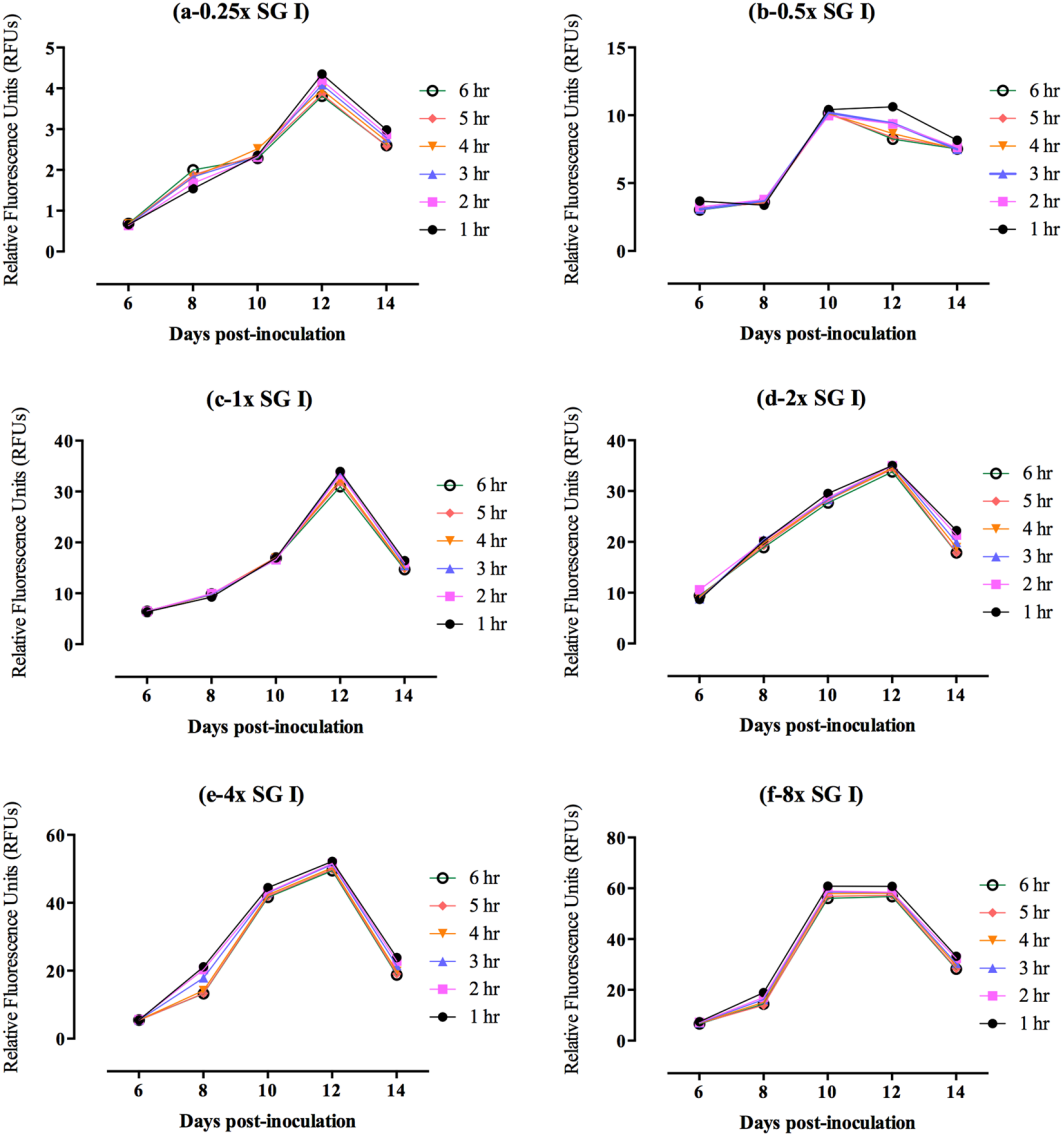
Optimization of the best condition of SYBR Green I (SG I) staining for fluorescence assay in mice using different concentrations of the stain. (**a**) 0.25x SG I; (**b**) 0.5x SG I; (**c**) 1x SG I; (**d**) 2x SG I; (**e**) 4x SG I; (**f**) 8x SG I. Each value represents the mean of duplicate trails of 5 mice per experimental group after subtracting the background fluorescence of non-parasitized RBCs from uninfected mice. Gain values are not set to 100.

**Figure 3:**
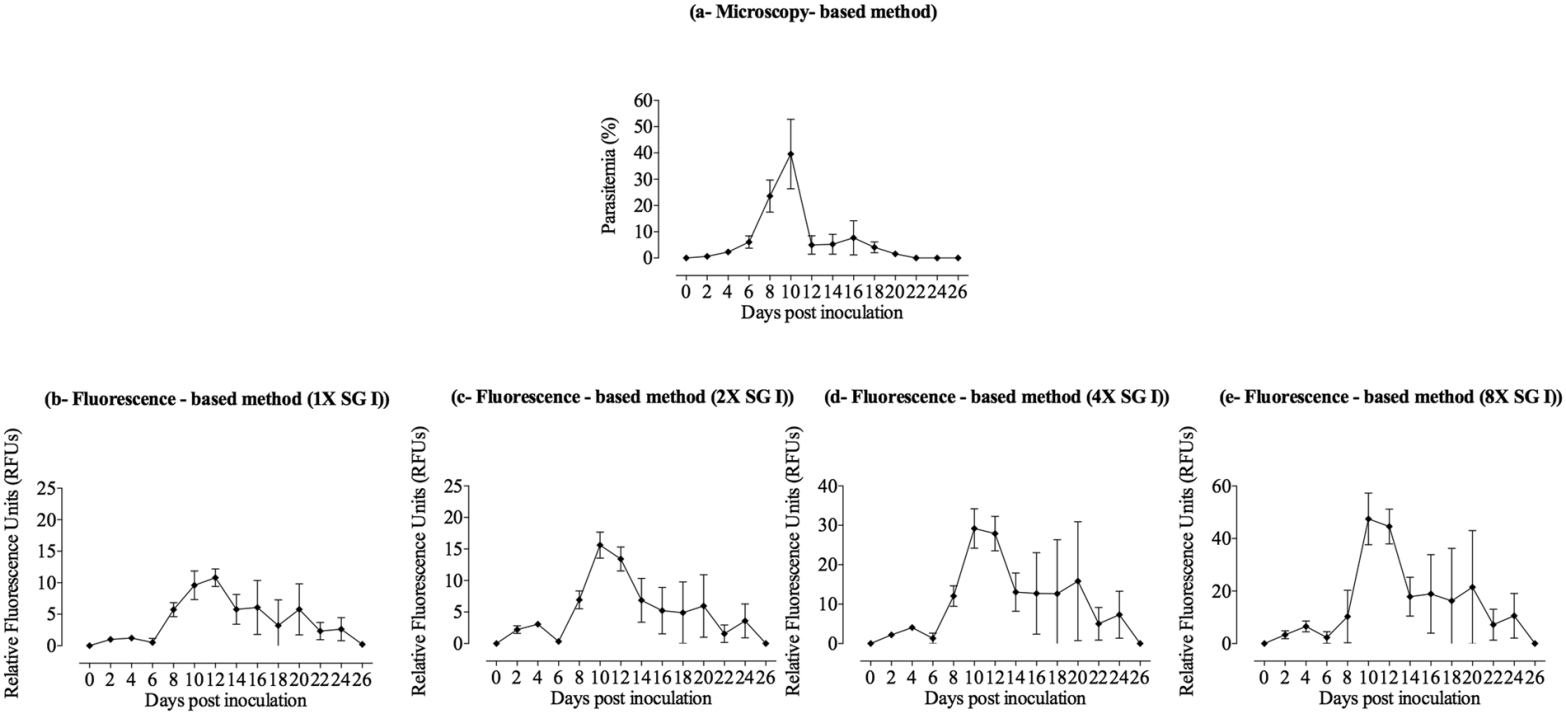
Comparison of the emitted fluorescence signals and parasitemia detected by fluorescence- and microscopy-based methods, respectively, using different concentrations of SYBR Green I stain. (**a**) Microscopy-based method; (**b**) fluorescence-based method using 1x SG I stain; (**c**) fluorescence-based method using 2x SG I stain; (**d**) fluorescence-based method using 4x SG I stain; (**e**) fluorescence-based method using 8x SG I stain. Each value represents the mean ± standard deviation of 5 mice per experimental group after subtracting the background fluorescence for non-parasitized RBCs from uninfected mice. Gain values are not set to 100.

To determine the best concentration of SG I stain for differentiating between infected and noninfected RBCs and which optimal period is sufficient for the complete interaction of SG I stain and parasite DNA, 2.5-µl blood samples were collected from noninfected mice and *B. microti*-infected mice on days with peak parasitemia and were mixed with lysis buffers containing different concentrations of SG I stain. The results revealed that 1 hour of incubation is sufficient for complete interaction between the stain and *B. microti* DNA; the differences between noninfected and infected RBCs at days 6, 8, 10, 12, and 14 post-inoculation were found to be optimal when stained with 1x or greater concentrations of SG I stain (Fig. 2). Therefore, the experiment was repeated in *B. microti-*infected mice using different concentrations of SG I greater than 1x; the fluorescence readings were obtained after a 1-hour incubation period and compared with the parasitemia monitored by the microscopy method. The results showed that peak parasitemia and fluorescence signals were observed on the same days p.i. when using 2x or greater concentrations of SG I stain (Fig. 3), although 4x or 8x concentrations of SG I stain exhibited high background signals with high standard deviations as compared with 2x SG I stain concentration (Fig. 3). Consequently, 2x SG I stain is the optimal concentration suitable for antibabesial drug screening in mice using fluorescence assay.

### Effect of WBCs on SYBR Green I fluorescence in mice

**Figure 4:**
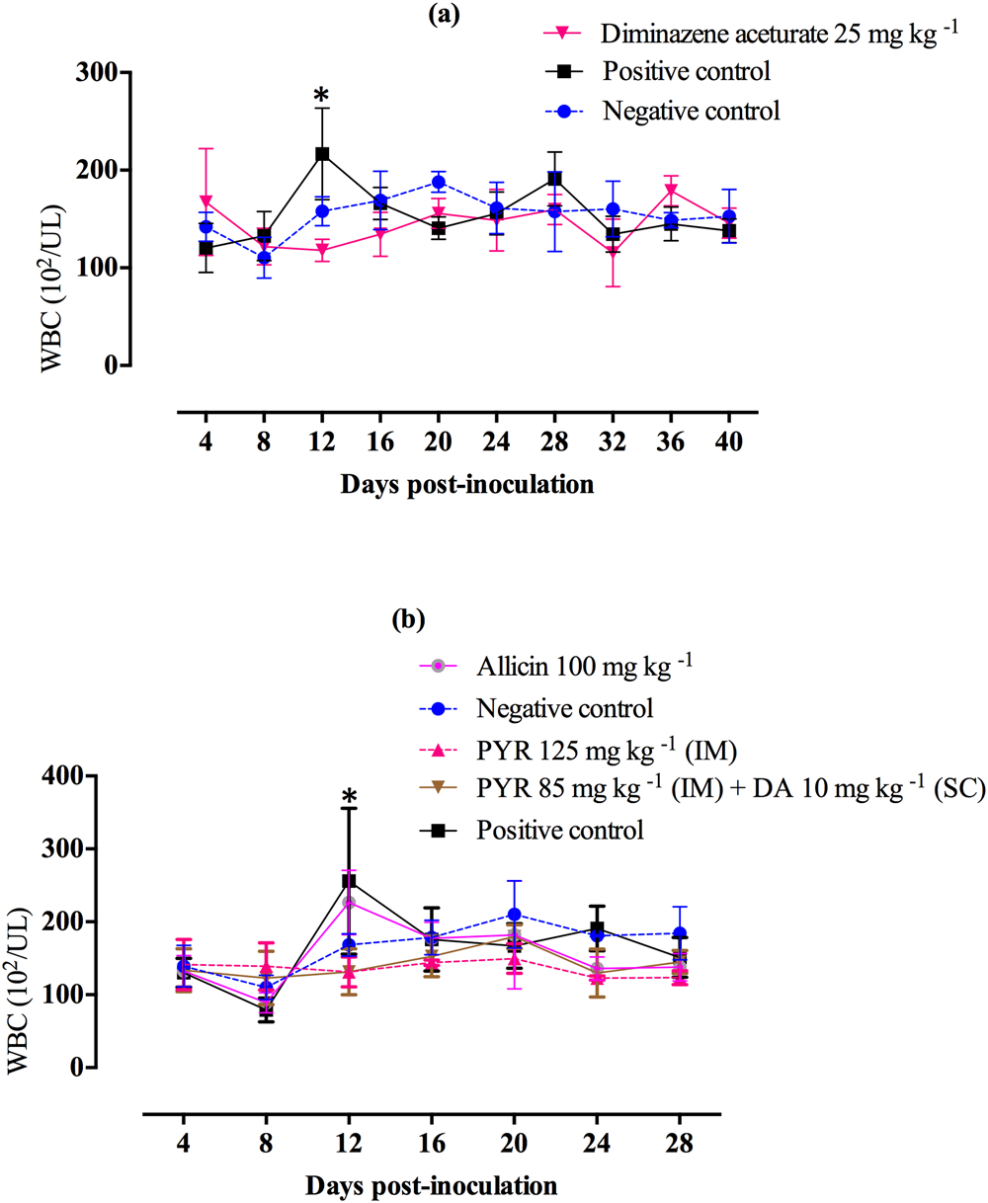
Changes in white blood cell (WBC) values in uninfected and treated *B. microti*-infected mice. (**a**) Mice treated with diminazene aceturate (DA) (25 mg kg^−1^); (**b**) mice treated with allicin (100 mg kg^−1^), pyronaridine tetraphosphate (PYR) (125 mg kg^−1^), and combination therapy of DA (10 mg kg^−1^) and PYR (85 mg kg^−1^). Each value represents the mean ± standard deviation of 5 mice per experimental group. Asterisks indicate a significant difference (*P* < 0.05) between treated or infected mice and uninfected mice.

Changes in WBC levels were monitored in *B. microti*-infected mice that served as positive control and those treated with DA, PYR, a PYR/DA combination, or allicin in three separate experiments in an attempt to evaluate the possible effect of WBCs in whole blood on SYBR Green I fluorescence signals. Statistically significant elevations (*P* < 0.05) were observed in mice treated with double-distilled water (DDW) (positive control) on a day with peak parasitemia 12 days post-inoculation (p.i.) as compared with negative control mice (Fig. 4a,b). On the contrary, no statistically significant differences (*P* > 0.05) were observed in WBC levels in all treated groups as compared with uninfected mice on all days p.i. (Fig. 4).

### Comparison of fluorescence- and microcopy-based methods

**Figure 5:**
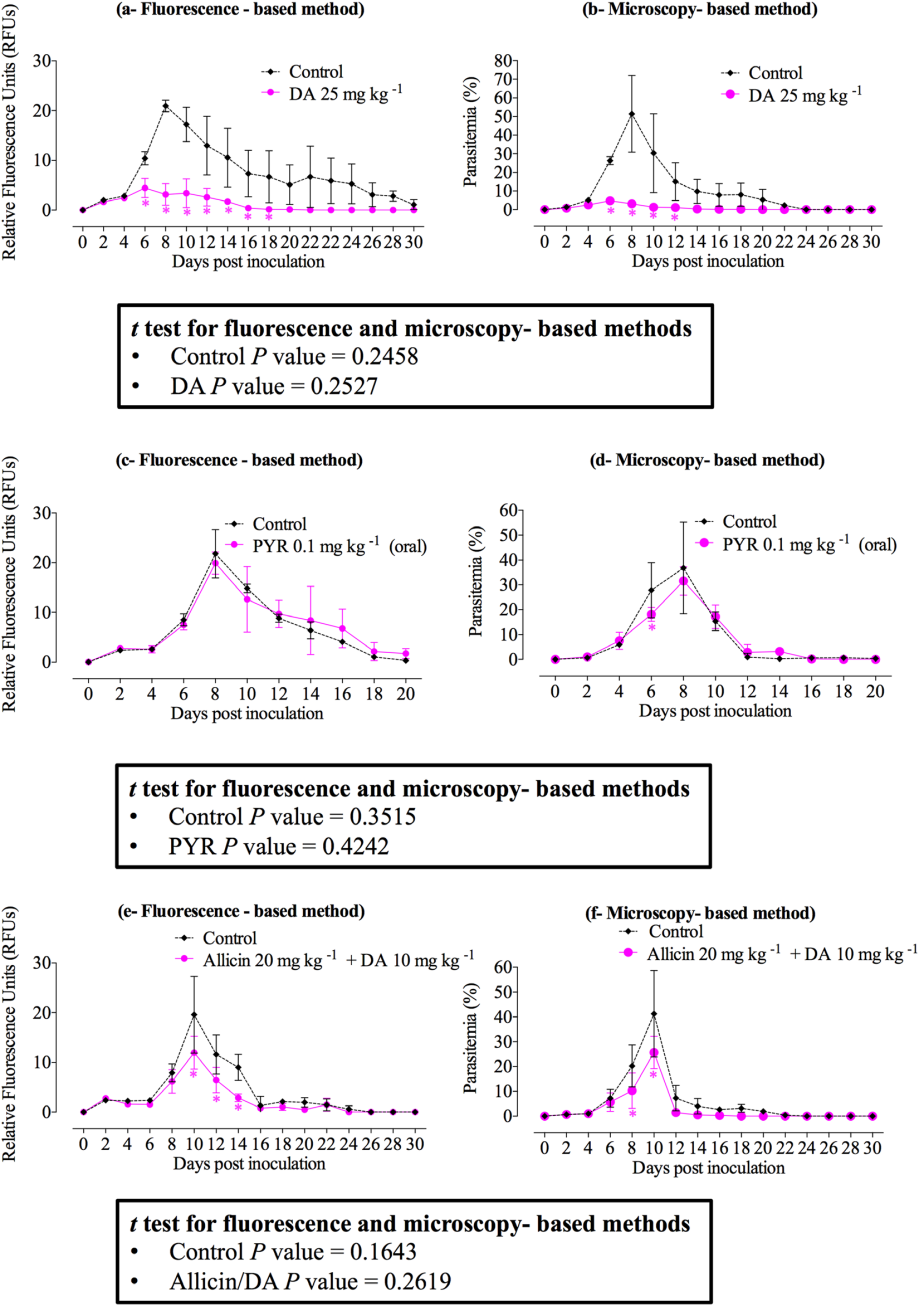
Comparison of the inhibitory effects of selected antibabesial drugs on the growth of *Babesia microti* estimated by microscopy- and fluorescence-based methods in uninfected and infected mice. (**a**) Inhibitory effect of diminazene aceturate (DA) estimated by fluorescence-based method; (**b**) inhibitory effect of diminazene aceturate (DA) estimated by microscopy-based method; (**c**) inhibitory effect of pyronaridine tetraphosphate (PYR) estimated by fluorescence-based method; (**d**) inhibitory effect of pyronaridine tetraphosphate (PYR) estimated by microscopy-based method; (**e**) inhibitory effect of allicin/DA combination estimated by fluorescence-based method; (**f**) inhibitory effect of allicin/DA combination estimated by microscopy-based method. Each value represents the mean ± standard deviation of 5 mice per experimental group after subtracting the background fluorescence for non-parasitized RBCs from uninfected mice. Asterisks indicate significant differences (*P < 0.05) between treated and control mice. Student’s *t*-test, *P* < 0.05 between control and treated groups by fluorescence- and microscopy-based methods. Gain values are not set to 100.

Evaluation of the fluorescence-based assay in mice required determining on which days there was a significant inhibition between the obtained parasitemia percentages and fluorescence signals in DA-, PYR-, and combination allicin/DA-treated mice and DDW- or normal saline-treated mice (positive control) using fluorescence- and microscopy-based methods at 2.5% HCT. Furthermore, detecting which day showed peak parasitemia percentages using a microscopy-based method and peak fluorescence values in treated mice using a fluorescence-based method was necessary for evaluating the assay’s usefulness for antibabesial drug screening in mice. The results revealed that fluorescence signals and parasitemia significantly decreased (*P* < 0.05) in DA-treated mice from days 6 to 18 p.i. and from days 6 to 12 p.i., respectively, as compared to those of controls (Fig. 5a,b). Moreover, mice treated with PYR showed significant decreases (*P* < 0.05) in parasitemia levels on day 6 p.i. as compared to those of controls using the microscopy method (Fig. 5d). On the contrary, no significant difference was detected in fluorescence signals in PYR-treated mice using the fluorescence method (Fig. 5c). Mice treated with an allicin and DA combination formula exhibited a significant decrease (*P* < 0.05) in fluorescence signals and parasitemia on days 10, 12, and 14 p.i. and on days 8 and 10 p.i., respectively, as compared to controls (Fig. 5e,f). Fortunately, both methods showed peak parasitemia and fluorescence signals on the same days p.i. in positive control mice and those treated with DA, PYR, and allicin combined with DA (Fig. 5). In the DA-treated group, control mice showed peak fluorescence signals of 20.94 ± 1.18 and peak parasitemia percentages of 51.42 ± 20.64 at 8 days p.i., whereas DA-treated mice showed peak fluorescence signals of 4.47 ± 1.92 and peak parasitemia of 4.78 ± 0.03 at 6 days p.i. (Fig. 5a,b). Additionally, in the PYR-treated group, control mice showed peak fluorescence signals of 21.78 ± 4.86 and peak parasitemia percentages of 36.82 ± 18.46 8 days p.i., while PYR-treated mice showed peak fluorescence signals of 19.91 ± 2.24 and peak parasitemia of 31.58 ± 5.71 8 days p.i. (Fig. 5c,d). Mice receiving intraperitoneal (IP) treatment with an allicin and DA combination showed peak fluorescence signals of 11.96 ± 3.29 and peak parasitemia percentages of 25.70 ± 6.50 10 days p.i., whereas control mice in the same group exhibited peak fluorescence signals of 19.60 ± 7.71 and peak parasitemia of 41.23 ± 17.39 10 days p.i. (Fig. 5e,f). These results demonstrate the usefulness of the fluorescence-based assay for drug screenings in mice infected by the *B. microti* parasite at 2.5% HCT.

### Confirming the validity of *Babesia* fluorescence assay (*B*FA) in mice

**Figure 6:**
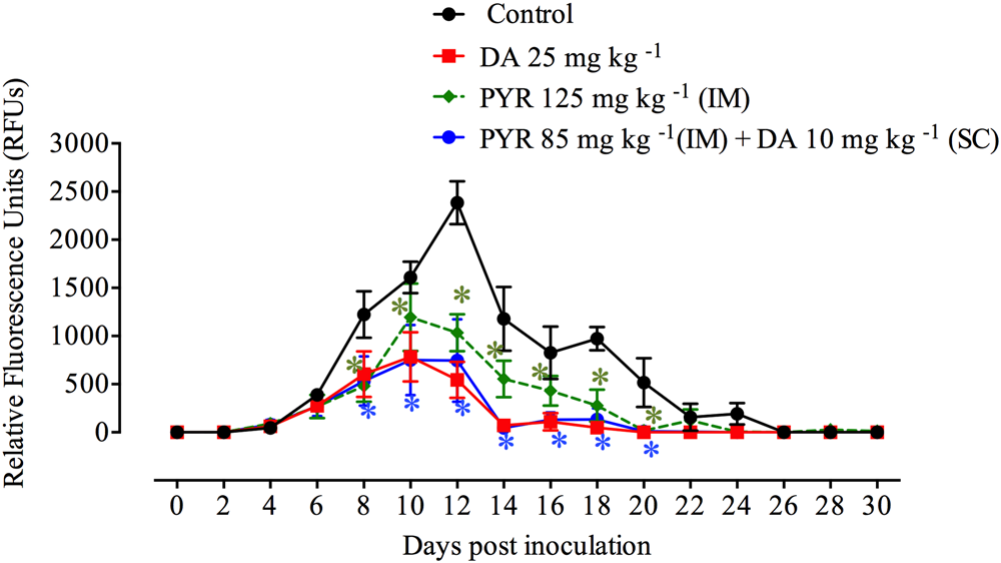
Inhibitory effect of diminazene aceturate (DA), pyronaridine tetraphosphate (PYR), and combination therapy of DA and PYR on the growth of *Babesia microti* as estimated by the fluorescence-based method. Each value represents the mean ± standard deviation of 5 mice per experimental group after subtracting the background fluorescence for non-parasitized RBCs from uninfected mice. Asterisks indicate significant differences (*P < 0.05) between treated and control mice. Gain values are set to 100.

We assessed the fluorescence values in *B. microti*-infected mice exposed to two drugs (DA and PYR) from day 2 to day 30 p.i. using *B*FA to confirm the assay’s usefulness for antibabesial drug screening in mice. Control mice treated with DDW exhibited rapid growth in the parasitemia level with a corresponding fluorescence value of 2382.98 on day 12 p.i. On the contrary, mice treated with DA or PYR separately showed peak fluorescence values of 783.50 and 1194.16, respectively, on day 10 p.i. (Fig. 6). In an astonishing way, the PYR/DA combination was effective against the growth of *B. microti*, with peak fluorescence values of 751.63 on day 10 p.i. Furthermore, as compared to control mice, the significant inhibition (*P* < 0.05) of fluorescence signals on days 8–20 p.i. was obtained in the presence of 10 mg kg^−1^ of DA combined with 85 mg kg^−1^ PYR (Fig. 6). Additionally, treating mice with this combination formula resulted in 56.35%, 53.25%, and 68.69% inhibition on days 8, 10, and 12 p.i., respectively. Notably, mice treated with the combined formula exhibited significant lower fluorescence values as compared to those treated with 25 mg kg^−1^ of DA or 125 mg kg^−1^ PYR separately (Fig. 6). These results confirmed the successful application of *B*FA for antibabesial drug screenings against *B. microti* in mice. In addition, the combination of PYR and DA effectively inhibited *Babesia* parasites *in vivo*.

### Role of PYR/DA combination on the regression of anemia in treated mice

**Figure 7:**
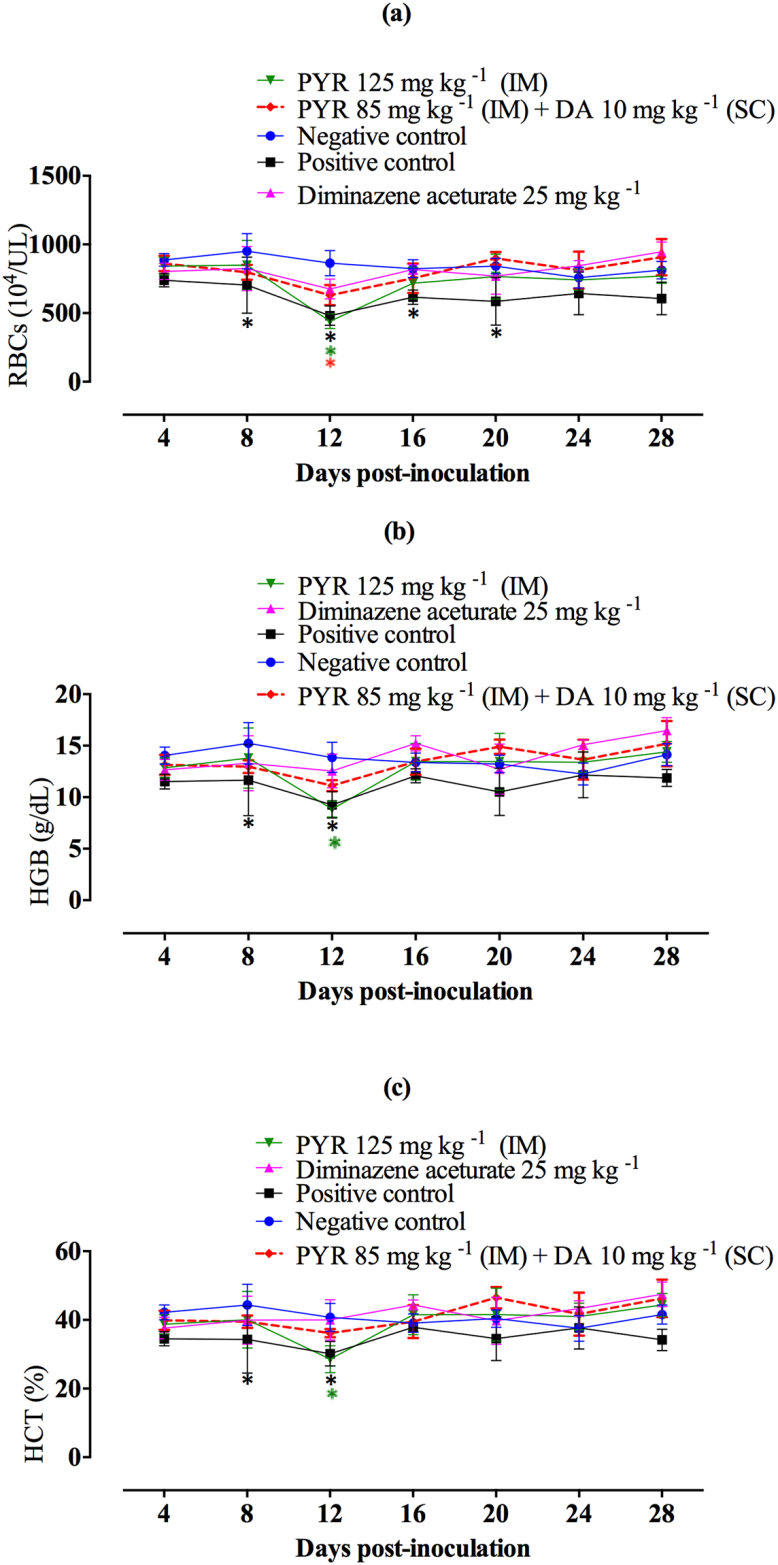
Anemia monitoring in mice treated by diminazene aceturate (DA), pyronaridine tetraphosphate (PYR), and a combination of both drugs. (**a**) RBC counts; (**b**) hemoglobin (HGB) levels; (**c**) hematocrit values. Each value represents the mean ± standard deviation of 5 mice per experimental group. Asterisks indicate a significant difference (*P* < 0.05) between treated or infected mice and uninfected mice.

The role of a single dose of PYR alone or in combination with DA on the retrogression of anemia was evaluated by monitoring the changes in HCT values, hemoglobin (HGB) levels, and red blood cell (RBC) counts. A significant reduction (*P* < 0.05) in RBC counts was observed in infected mice that received DDW (positive control) on days 8, 12, 16, and 20 after infection as compared with uninfected mice (Fig. 7a). Additionally, significant reductions (*P* < 0.05) in RBC counts were observed in mice treated either with PYR alone or a PYR/DA combination as compared with uninfected mice on day 12 p.i. (Fig. 7a).

Significant reductions (*P* < 0.05) of HGB and HCT levels were observed in infected mice and served as a control on days 8 and 12 (Fig. 7b,c). Moreover, mice treated with a single intramuscular dose of PYR alone showed significant reductions in HGB and HCT levels on day 12 p.i. (Fig. 7b,c). On the contrary, no significant reduction (*P* > 0.05) was observed in either HGB and HCT levels in mice treated with a PYR/DA combination as compared with uninfected mice on all days p.i. (Fig. 7b,c). The results indicate a quick recovery from anemia in mice treated with a PYR/DA combination and confirmed the potential antibabesial effect of this combination therapy.

### Detection of parasite nucleic acid remnants in the organs of treated mice

**Figure 8:**
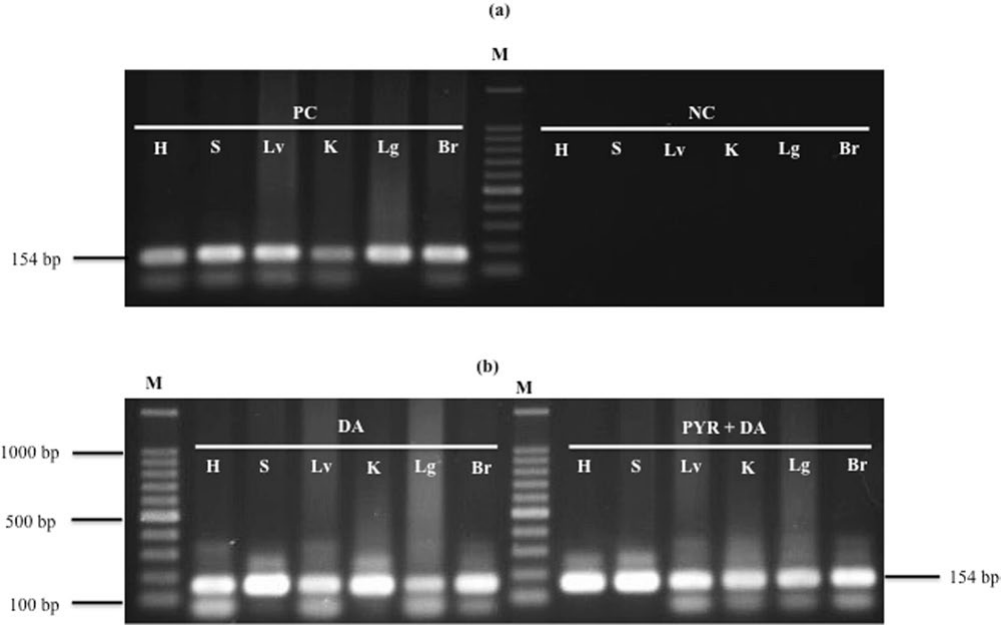
PCR of the ss-rRNA gene in different organs of mice on day 30 post-infection. (**a**) *Babesia microti-* infected mice that received no treatment (positive control) and non-infected mice (negative control). (**b**) mice infected with *Babesia microti* after treatment with 25 mg kg^−1^ of diminazene aceturate (DA) and a pyronaridine tetraphosphate (PYR) (85 mg kg^−1^)/DA (10 mg kg^−1^) combination. PC, positive control; NC, negative control; Br, brain; Lg, lung; K, kidney; Lv, liver; S, spleen; H, heart; M indicates a 100-bp DNA ladder. The expected size of the PCR product was 154 bp. Full-length gels are presented in Supplementary Figure S1.

Weak emitted fluorescence signals were obtained from the blood samples collected from *B. microti*- infected mice treated with PYR/DA combination therapy using *B*FA on day 30 post-infection. These signals were nearly similar to those obtained from uninfected mice (negative control), indicating the potential antibabesial effect of this combination therapy. As the fluorescence-based assay was simple and rapid to examine parasite DNA in the blood compared with PCR, the blood of infected mice in the present study was not used in PCR for the detection of parasite DNA. PCR is time consuming since the technique involves DNA extraction, amplification of a DNA fragment, and electrophoresis of the fragment. Blood samples mixed with SYBR Green I produced fluorescence signals in proportion with parasite DNA concentration in RBC. The intensity of emitted fluorescence signals from blood samples of infected mice were good indicators to the *Babesia* DNA concentrations within the RBCs. In stead, a fluorescence-based assay can’t be used for other organs to detect parasite DNA since different kinds of tissue cells were included in the organs and SYBR Green I will bind to not only parasite DNA but also to tissue cells DNA that cause the emission of high nonspecific signals. Subsequently, the fluorescence-based assay is not specific for *Babesia* in the organs of infected mice. Therefore, PCR was applied for DNA samples extracted from different organs collected from mice on day 30 p.i. to detect the ability of the administrated antibabesial hits to further eradicate the parasite nucleic acid from the animal’s body. Unfortunately, PCR amplification detected *B. microti* ss-rRNA genes in the spleen, heart, liver, kidneys, lung, and brain of mice treated either with DA alone or a PYR/DA combination on day 30 p.i. (Fig. 8). These results indicate the incompetence of either DA alone or DA combined with PYR to completely eliminate *B. microti* nucleic acid from the animal’s body on day 30 p.i., consequently, reinfection is suspected.

## Discussion

Diminazene aceturate and imidocarb dipropionate are widely used as antibabesial drugs for the treatment of infected animals; however, the emergence of drug resistance and toxicity^4^ make the search for new, more effective, and safer antibabesial agents imperative. Such a search requires the development of a novel method that will overcome the drawbacks of the microscopy method traditionally used. Therefore, this study evaluated the validation and consistency of fluorescence assay for antibabesial drug screening against *B. microti* in specific pathogen-free mice. Recently, large-scale drug screening against the *in vitro* growth of *B. bovis*, *B. bigemina*, *B. caballi*, *T. equi*, *B. divergens*, and *P. falciparum* parasites was established using a fluorescence-based method with SG I^9–12^. The quality of the assay depends on the values of the HTS assay statistical parameters (Z′ factor, S/N ratio, % CV_max_, and % CV_min_)^13–15^. Collectively, Z′ factor values higher than 0.5 along with the highest S/N ratio value revealed the strength and precision of the assay in generating trustworthy data^16^. However, we established the assay *in vitro*; this is the first study to evaluate the usefulness of fluorescence assay for antibabesial drug screening in mice.

To establish the assay in specific pathogen-free mice, the correlation between emitted fluorescence signals and parasitemia percentages, in addition to the HTS assay, was assessed with two different HCTs (2.5% and 5%). Next, the fluorescence values were determined using different concentrations of SG I stain after different periods of incubation to determine the best concentration for differentiating between infected and noninfected RBCs and determining the optimal period for complete interaction between the SG I stain and parasite DNA. After that, changes in the WBC levels were monitored in noninfected and *B. microti*-infected mice in an attempt to evaluate the possible effect of WBCs in whole blood on SYBR Green I fluorescence signals. Thereafter, microscopy- and fluorescence-based methods were compared using the best HCT identified from linearity and HTS assay. In all experiments in this study, SYBR Green I was mixed with RPMI 1640 Medium, due to the stable high background obtained with this medium relative to distilled water^17^. Moreover, half the amount of lysis buffer without SG I stain was first mixed with the medium to help RBCs hemolysis and prevent blood coagulation. Then, the remaining amount of lysis buffer containing SG I stain was added to each well of a 96-well plate.

The correlation between the emitted fluorescence signals and parasitemia percentages showed significant linear relationships at both HCTs used. These results were in accordance with those previously obtained for bovine and equine *Babesia* and *Theileria* parasites *in vitro* ^9^. The HTS assay results with 2.5% HCT generated Z′ factor values within the permissible limit and the highest S/N ratios on all days with peak parasitemia, which was in agreement with previously obtained values of bovine *Babesia* parasites^9^. Additionally, the assay with 2.5% HCT revealed S/N ratios 2–11, 2–3, 3–8, 28–130, 187–1375, and 612–1215 times higher than those obtained from *B. bovis*, *B. bigemina*, *B. divergens*, *P. falciparum*, *T. equi*, and *B. caballi* research *in vitro*, respectively^9,10,17^.

SYBR Green I is a highly sensitive indicator of DNA. However, it is an unspecific stain, due to its poor ability to discriminate between parasitic and nonparasitic DNA, such as WBCs or other microorganisms. Therefore, in this study, WBC levels were monitored in mice treated with DA, PYR, PYR/DA, or allicin. Significant elevations (*P* < 0.05) were observed in positive control mice on days with peak parasitemia, as compared with uninfected (negative control) mice. The stability of WBC levels as compared with those of uninfected mice indicated the great potential of *B*FA for drug screening in controlled laboratory mice (specific pathogen free, same age and gender) because of its high speed and relatively low cost. However, other studies are required before the assay can be applied in clinical cases in the field, due to the strong background signals of WBCs that may be observed in such cases. Regarding this issue, a complete removal of WBCs from whole blood samples may be applied using either Whatman columns or commercially available leukocyte filters.

The results obtained from linearity, HTS assay, and by using different concentrations of SG I indicated the usefulness of *B*FA at 2.5% HCT and 2x SG I stain. Therefore, DA, PYR, and an allicin/DA combination were used to evaluate the assay for antibabesial drug screening in mice infected by *B. microti* in these optimal conditions. There was a minor difference in the days showing significant inhibition of parasitemia levels and fluorescence values in mice treated by microscopy- and fluorescence-based methods, respectively. However, peak parasitemia and fluorescence values were observed on the same days p.i., either in positive control mice or treated mice. Of note, no significant differences (*P* > 0.05) were detected between fluorescence- and microscopy-based methods, either in treated or non-treated mice on all days. Indeed, the microscopy-based method cannot detect remnants of parasite nucleic acid in mouse blood. On the other hand, SG I stain is highly sensitive in detecting parasite nucleic acid, resulting in higher fluorescence values as compared with the parasitemia levels detected by the microcopy method. Therefore, slight differences in the days exhibiting significant inhibition of the parasitemia level by the microscopy-based method and fluorescence values by the fluorescence-based method were observed in treated mice in this study. The consistency in the days with peak parasitemia and fluorescence values and the stability of the days showing significant inhibition as compared with controls by microscopy- and fluorescence-based methods indicate the robustness of *B*FA for antibabesial drug screenings in mice infected by *B. microti* at 2.5% HCT.

To confirm the usefulness of *B*FA for screening large chemical libraries in mice infected by *B. microti*, DA and PYR drugs were used in three separate experiments. Pyronaridine tetraphosphate had not yet been tested against *B. microti* in mice; however, its efficacy against the *in vitro* growth of *B. bovis*, *B. bigemina*, *T. equi*, *B. caballi*, *B. divergens*, and *P. falciparum* has previously been proven^9,10,18^, which encouraged us to further evaluate its *in vivo* effects on *B. microti* in a mouse model. Furthermore, in an attempt to overcome resistance to DA and reduce its side effects, we examined the inhibitory effects of PYR combined with low doses of DA. The inhibitory effects caused by 10 mg kg^−1^ of DA combined with 85 mg kg^−1^ PYR on *B. microti* growth were higher than those previously determined using the microscopy method for epoxomicin treatment (0.05 and 0.5 mg kg^−1^), which were 36.3 and 47.6% inhibition, respectively, on day 10 p.i.^19^. The inhibitory effects of this combined therapy on *B. microti* growth were similar to those caused by nerolidol (100 mg kg^−1^), which showed 53.7% inhibition on day 10 p.i.;^20^ clindamycin (500 mg kg^−1^), which showed 68.5% inhibition on day 7 p.i.;^6^ and clindamycin combined with quinine, which demonstrated 70% inhibition in the growth of *B. microti* on day 7 p.i.^21^.

Interestingly, this combined therapy prevented the development of anemia, as there was no significant reduction in HCT and HGB levels in treated mice on all days p.i. Although PYR/DA combined therapy showed significant reductions in RBC counts, the reduction was transient and observed only on the day with peak parasitemia. Moreover, the combined therapy exhibited rapid recovery of HCT levels as compared with those recently observed with clofazimine, which causes significantly lower hematocrit values on days 8 and 12 p.i. than those of the control^22^. Although the PYR/DA combination exhibited potential antibabesial effect against *B. microti* in the blood collected from treated mice using fluorescence assay, parasite’s DNA was detected in the mice organs by nested PCR 30- days post-treatment. Amplification of parasite’s DNA fragment that extracted from the mice’s organs by nested PCR, while determination of parasite’s nucleic acid in mice blood using fluorescence assay based on SG I stain, may explain such discrepancy. Furthermore, the short period of the experiment (30 days) may explain the presence of DNA remnants in mice organs. Therefore, future studies are required to evaluate the effect of PYR in combination with either DA or other antibabesial drugs, such as (−)-epigallocatechin-3-gallate^23^ or allicin^7^, for a time more prolonged than that used in the current experiment.

Azithromycin with atovaquone or clindamycin with quinine are combined therapies used currently to treat human babesiosis. However, due to the development of drug resistant parasites coinciding with therapeutic failure in severe cases, they have not been used consistently^2,24^. Therefore, PYR as presented in this study might be an alternative chemotherapeutic agent against human babesiosis caused by *B. microti*. Since PYR is being used in combination with artesunate to treat malaria^25^, it has potential for treating *B. microti* in humans as an alternative to currently used drugs.

This study opens the door for using more rapid, robust, and automatic assays than the microscopy method traditionally used for antibabesial drug screening in mice kept under specific pathogen-free conditions. In fact, hemoparasitic diseases are associated with the efflux of erythroblasts and normoblasts into the blood circulation in cases associated with severe anemia. These nucleated cells can be stained by DNA/RNA dyes and can be confused with infected red blood cells (IRBCs)^26^. Thus, further studies are required to assess the validity of *B*FA in cases associated with severe anemia, as in mice infected with *B. rodhaini* or severely affected clinical cases in field conditions. In such cases, we must also examine the assay’s ability to assess white blood cell and reticulocyte frequencies in parallel with parasitemia.

Because all tests were done under optimal laboratory conditions, we concluded that *Babesia* fluorescence assay at 2.5% HCT for *B. microti* parasites offers a novel approach for the screening of chemical libraries in mice. However, in clinically affected cases in field conditions, this assay seems to show great potential for rapid drug screening when WBC and reticulocyte-free cultures can be obtained. Additionally, the present study demonstrated the potential inhibitory effect of a DA and PYR combination against *B. microti* parasite, suggesting that this drug combination might be promising for the treatment of clinical diseases caused by *Babesia* and *Theileria* in animals. These findings warrant further investigation to evaluate the possible use of PYR in combination with other drugs for animal piroplasmosis and human babesiosis.

## Methods

### Ethics statement

All experimental protocols in this study were approved by the Animal Care and Use Committee, Obihiro University of Agriculture and Veterinary Medicine (Approval No. 27–65). All experiments were conducted in accordance with the Fundamental Guidelines for Proper Conduct of Animal Experiment and Related Activities in Academic Research Institutions under the jurisdiction of the Ministry of Education, Culture, Sports, Science and Technology, Japan.

### Parasites and mice

Female BALB/c mice aged 8 weeks were purchased from CLEA Japan (Tokyo, Japan) for use in this study and kept under specific pathogen-free conditions. The Munich strain of *B. microti* was maintained by passage in the blood of BALB/c mice^22^.

### Chemical reagents

SYBR Green I (SG I) nucleic acid stain (Lonza, USA; 10,000x) was stored at −20 °C and thawed before use. A lysis buffer consisting of Tris (130 mM; pH 7.5), Ethylenediaminetetraacetic acid (EDTA) (10 mM), saponin (0.016%; W/V), and TritonX-100 (1.6%; V/V) was prepared in advance and stored at 4 °C. Diminazene aceturate (Ciba-Geigy Japan Limited, Japan), Pyronaridine tetraphosphate (Sigma-Aldrich Japan) and allicin (iHerb.com) were used in this study to evaluate the validity of fluorescence-based assays for antibabesial drug screening in mice.

### Assessment of SYBR Green I fluorescence linearity

Two female BALB/c mice were used to assess the linearity between the fluorescent values and parasitemia as determined by microscopy. One mouse was inoculated intraperitoneally with 1 × 10^7^ *B. microti*-infected RBCs, and the other mouse remained uninfected. The level of parasitemia in the infected mouse was monitored daily using Giemsa-stained thin blood smears prepared from venous tail blood, and when the parasitemia reached 60%, the linearity was assessed. A 96-well plate was used to serially dilute the *B. microti* pRBCs with non-parasitized RBCs from the uninfected mouse to parasitemia ranging from 0.25% to 50% in 100 uL^27^. Non-parasitized mice RBCs from the uninfected mouse were used as a blank control. A thin blood smear was also prepared from each dilution and stained with Giemsa to confirm the parasitemia by microscopy. Mice RBCs were prepared in RPMI 1640 Medium^16,28^ containing 50 µl of a lysis buffer in triplicate at two different hematocrits (HCTs) (2.5% and 5%). Next, 50 µl of a lysis buffer mixed with a 2 × SG I (10,000x) nucleic acid stain was added directly to each dilution by gentle mixing. Afterward, plates were incubated for 6 hours in a dark place at room temperature, and fluorescence values were determined using a fluorescence plate reader (Fluoroskan Ascent, Thermo Labsystems, USA) at 485 nm and 518 nm excitation and emission wavelengths, respectively. Gain values were set to 100. The parasitemia (x value) was plotted against the RFU (y value) after background subtraction of non-parasitized mouse RBCs and analyzed by linear regression. The experiment was repeated three times.

### Determination of statistical parameters for high-throughput screening (HTS) assay

Fifteen female BALB/c mice were used to determine the quality of HTS assay in three separate trials. The mice were divided into three groups, each containing five mice. The first and second groups were inoculated intraperitoneally with 1 × 10^7^ *B. microti*-infected RBCs, while the third group remained uninfected and was used as a blank control. The level of parasitemia in the infected mice was monitored daily using stained thin blood smears, and when the parasitemia reached 1%, mice in the first group were administered daily subcutaneous injections of DA at a high nonlethal dosage, 70 mg kg^−1^ ^29^, for 5 successive days (negative growth). The second group remained without treatment and was used as a positive control (positive growth, 100% growth). The levels of parasitemia in all mice were monitored daily until the peak of parasitemia was detected by examination of stained thin blood smears prepared from venous tail blood. On days 5, 6, and 7 (peak of parasitemia) p.i., the quality of the HTS assay was determined using a 96-well plate, as follows. Non-parasitized RBCs from uninfected mice were used as a blank control. Venous tail blood samples of 2.5 µl and 5 µl were collected from each mouse in RPMI 1640 Medium previously mixed with 50 µl of a lysis buffer in triplicate at two different HCTs (2.5% and 5%). Next, 50 µl of a lysis buffer containing 2 × SG I nucleic acid stain was added directly to each well and gently mixed. Thereafter, the plate was incubated, and fluorescence values were determined as previously mentioned. Statistical parameters, including the Z′ factor (Z′), signal-to-noise (S/N) ratio, coefficient of variation at the maximum signal (% CV_max_, positive control), and coefficient of variation at the minimum signal (% CV_min_, negative control), were calculated to determine the assay quality, in accordance with the standards of Zhang *et al*.^15^^.^

### Establishment of optimal SYBR Green I staining for fluorescence assay

To determine the optimal concentration of SYBR Green I and the optimal incubation time for discriminating between infected and noninfected RBCs with minimal background noise from WBCs, ten female BALB/c mice were used in three separate experiments. The mice were divided equally into two groups. The first group was inoculated intraperitoneally with 1 × 10^7^ *B. microti*-infected RBCs, while the second group remained uninfected and was used as a blank control. SYBR Green I concentrations ranging from 0.25 × to 8 × were used to stain RBCs from *B. microti*-infected mice at 2.5% HCT. The levels of parasitemia in the infected mice were monitored daily by microscopic examination of Giemsa-stained thin blood smears prepared from venous tail blood to determine the days with peak parasitemia. On days showing peak parasitemia, fluorescence analysis was performed after the samples were incubated in the dark at room temperature for 1, 2, 3, 4, 5, and 6 hours. Next, the experiment was repeated in another ten female BALB/c mice divided equally into 2 groups. The first group remained without infection and was used as a negative control, while the second group was intraperitoneally inoculated with 1 × 10^7^ *B. microti*-infected RBCs and used as a positive control. The emitted fluorescence signals were monitored every two days until the cessation of parasitemia by the *Babesia* fluorescence–based assay using 1x, 2x, 4x, and 8x SG I nucleic acid stain at 2.5 % HCT and compared with the levels of parasitemia in infected mice as detected by the microscopy method through the examination of 1000 erythrocytes in Giemsa-stained thin blood smears prepared from venous tail blood. The plates were incubated for 1 hour, and fluorescence values were determined as formerly described. The experiment was repeated two times.

### White blood cells’ (WBCs) effect on emitted fluorescence signals in treated mice

To assess the influence of WBCs in whole blood on SYBR Green I stain sensitivity, WBC levels were monitored in *B. microti*-infected mice treated with DA (dose rate 25 mg kg^−1^), PYR (dose rate 125 mg kg^−1^), a PYR/DA combination (dose rate 85 mg kg^−1^ and 10 mg kg^−1^), or DDW. Additionally, WBC levels were monitored in mice that remained without infection that served as a negative control. Moreover, to assess the possible effect of immunostimulant drugs on the levels of WBCs and emitted fluorescence signals, WBC levels were checked in mice treated with an immunostimulant drug as allicin (dose rate 100 mg kg^−1^). Five mice were used for each group. For each specific drug, mice were inoculated intraperitoneally with 1 × 10^7^ *B. microti*-infected RBCs. The drugs were administrated when the level of parasitemia in infected mice was checked microscopically and found to reach 1%. DA was administered subcutaneously; allicin was administrated intraperitoneally for 5 successive days, while for PYR, a single dose was administrated intramuscularly. Monitoring occurred every 96 h in three separate experiments using the Celltac α MEK-6450 automatic hematology analyzer (Nihon Kohden Corporation, Tokyo, Japan).

### Evaluation of the fluorescence-based assay *in vivo*

The *in vivo* growth inhibition assay was performed using microscopy and fluorescence-based methods. The microscopy method was applied according to the method previously described^30,31^, with some modifications. The inhibitory effects of DA, PYR, and allicin in combination with DA upon *B. microti* were used to evaluate the fluorescence assay for antibabesial drug screening of mice. Fifteen female BALB/c mice were used for each specific drug and divided equally into three groups. For each specific drug, mice in the first and second groups were inoculated intraperitoneally with 1 × 10^7^ *B. microti*-infected RBCs, while mice in the third group remained uninfected and were used as blank controls. The drug was administrated when the level of parasitemia in the infected mice, checked microscopically, was found to reach 1%. Mice in the first group were treated with specific drugs—DA, PYR alone, or allicin in combination with DA. Allicin was dissolved in 0.06% DMSO and resuspended in 0.1 ml of double-distilled water^7^. DA (10 mg kg^−1^) was dissolved in 0.1 ml of normal saline (NaCl 0.9%) and administrated to the infected mice intraperitoneally in the same inoculation period with allicin (20 mg kg^−1^) for 5 successive days. Meanwhile, PYR was dissolved in 0.1 ml of DDW, and a single dose from it was administered orally at a dose rate of 0.1 mg kg^−1^. DA (as a reference drug control) was administrated subcutaneously at a dose rate of 25 mg kg^−1^ for 5 successive days. DDW alone or DDW containing DMSO was administered to the second group as a placebo control^6^. The levels of parasitemia in all infected mice were monitored every two days until 20 days post-inoculation or the cessation of parasitemia by examination of 1000 erythrocytes in Giemsa-stained thin blood smears prepared from venous tail blood and the *B*FA using SG I nucleic acid stain. A 96-well plate was used. Non-parasitized RBCs from uninfected mice (third group) were used as a blank control. Venous tail blood samples of 2.5 µl were collected from each mouse in RPMI 1640 Medium previously mixed with 50 µl of a lysis buffer. Next, a lysis buffer containing 2x SG I nucleic acid stain was added directly to each well to complete 100 µl and gently mixed. Then, the plate was incubated, and fluorescence values were determined as formerly described. The experiment was repeated three times for each drug.

### Confirming the validity of *Babesia* fluorescence assay (*B*FA) in mice

To confirm the usefulness of *B*FA for mass drug screenings in mice, assays for DA and PYR were performed in BALB/c mice in three separate trials. Twenty-five female BALB/c mice were divided equally into five groups. Mice in the first four groups were intraperitoneally inoculated with 1 × 10^7^ *B. microti*-infected RBCs, while mice in the fifth group remained without infection and was used as a blank control. Parasitemia levels were monitored, and when the infected mice parasitemia reached approximately 1% in all infected groups, tested drugs were administrated. Mice in the first group received intramuscular nontoxic doses of PYR (125 mg kg^−1^), and mice in the second group were treated with antibabesial combination therapy consisting of 85 mg kg^−1^ single intramuscular doses of PYR and 10 mg kg^−1^ of subcutaneous doses of DA for 5 successive days. PYR and DA were dissolved in 0.05 and 0.1 ml of DDW, respectively, and administrated in the same inoculation period. DDW was administered subcutaneously^6^ to the mice in the fourth group, which was used as a placebo control. The emitted fluorescence signals were used as an indicator of the parasitemia percentages in all mice and monitored as previously mentioned every two days until the cessation of parasitemia.

### Role of PYR/DA combination on the regression of anemia associated with *Babesia* infection

HCT values, HGB levels, and RBC counts were used as indicators of the development of anemia in mice treated with PYR alone or combined with DA. To monitor anemia, blood samples (10 µl) were collected from each mouse that had received antibabesial drugs or that had received DDW and served as a positive control. Moreover, mice that remained without infection were used as negative controls. In all groups, the three anemia indicators were monitored every 96 h with the Celltac α MEK-6450 automatic hematology analyzer. The experiment was repeated three times.

### PCR detection of *B. microti* in treated mice

*B. microti* was detected in DNA samples from mouse tissues (heart, spleen, liver, kidney, lung, and brain) on day 30 p.i. using previously described diagnostic nested PCR assays targeting the *B. microti* small subunit rRNA (ss-rRNA) gene^32^. Primer sequencing and PCR cycling conditions were detailed in a previous report^24^. DNA was extracted from the tissue of *B. microti*-infected mice treated with DA (dose rate 25 mg kg^−1^), and a PYR/DA combination (dose rates 85 mg kg^−1^ and 10 mg kg^−1^) and the nontreated control (negative and positive) groups using the NucleoSpin Tissue Kit (Macherey-Nagel, Düren, Germany).

### Statistical analysis

Data was analyzed using a commercial statistical software program (GraphPad Prism version 5.0 for Windows; GraphPad Software, Inc., San Diego, CA, USA) using the independent Student’s *t*-test and a two-way ANOVA^31^. *P* < 0.05 was considered to be statistically significant for all tests.

### Data availability

The datasets generated during and/or analysed during the current study are available from the corresponding author on reasonable request.

## Electronic supplementary material

Supplementary Figure S1. Full-length gels for PCR of the ss-rRNA gene in different organs of mice on day 30 post-infection.
