# Supplementary material for: Performance and consistency of a fluorescence-based high-throughput screening assay for use in Babesia drug screening in mice
Source: Sci Rep. 2017 Oct 16;7:12774. doi: 10.1038/s41598-017-13052-5 (PMC5643553; doi:10.1038/s41598-017-13052-5)
Supplement: Supplementary file 1 — Supplementary Figure S1. Full-length gels for PCR of the ss-rRNA gene in different organs of mice on day 30 post-infection. [file 41598_2017_13052_MOESM1_ESM.pdf]

Title

Performance and consistency of a fluorescence-based high-throughput screening assay for use in *Babesia* drug screening in mice

Authors

Mohamed Abdo Rizk,[1,2] Shimaa Abd El-Salam El-Sayed,[1,3] Mahmoud AbouLaila,[1,4] Rasha Eltayesh,[1,5] Naoaki Yokoyama,[1] and Ikuo Igarashi[1]*

Addresses

[1] National Research Center for Protozoan Diseases, Obihiro University of Agriculture and Veterinary Medicine, Inada-Cho, Obihiro, Hokkaido 080-8555, Japan

[2] Department of Internal Medicine and Infectious Diseases, Faculty of Veterinary Medicine, Mansoura University, Mansoura 35516, Egypt

[3] Department of Biochemistry and Chemistry of Nutrition, Faculty of Veterinary Medicine, Mansoura University, Mansoura 35516, Egypt

[4] Department of Parasitology, Faculty of Veterinary Medicine, University of Sadat City, Sadat City 32511, Minoufiya, Egypt

[5] Department of Pharmacology, Faculty of Veterinary Medicine, Mansoura University, Mansoura 35516, Egypt

***Corresponding author**

Ikuo Igarashi, DVM, PhD

National Research Center for Protozoan Diseases, Obihiro University of Agriculture and Veterinary Medicine, Inada-Cho, Obihiro, Hokkaido 080-8555, Japan

Tel.: +81-155-49-5641; Fax: +81-155-49-5643; E-mail address: igarcpmi@obihiro.ac.jp

(a)

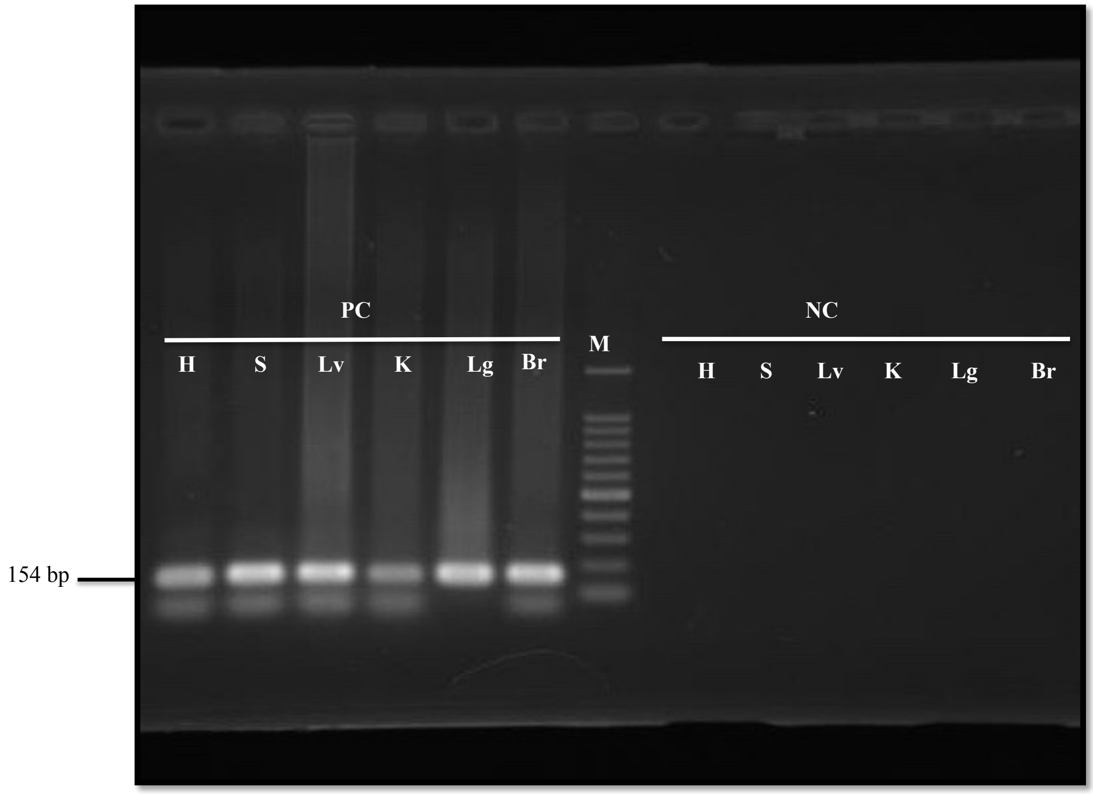

(b)

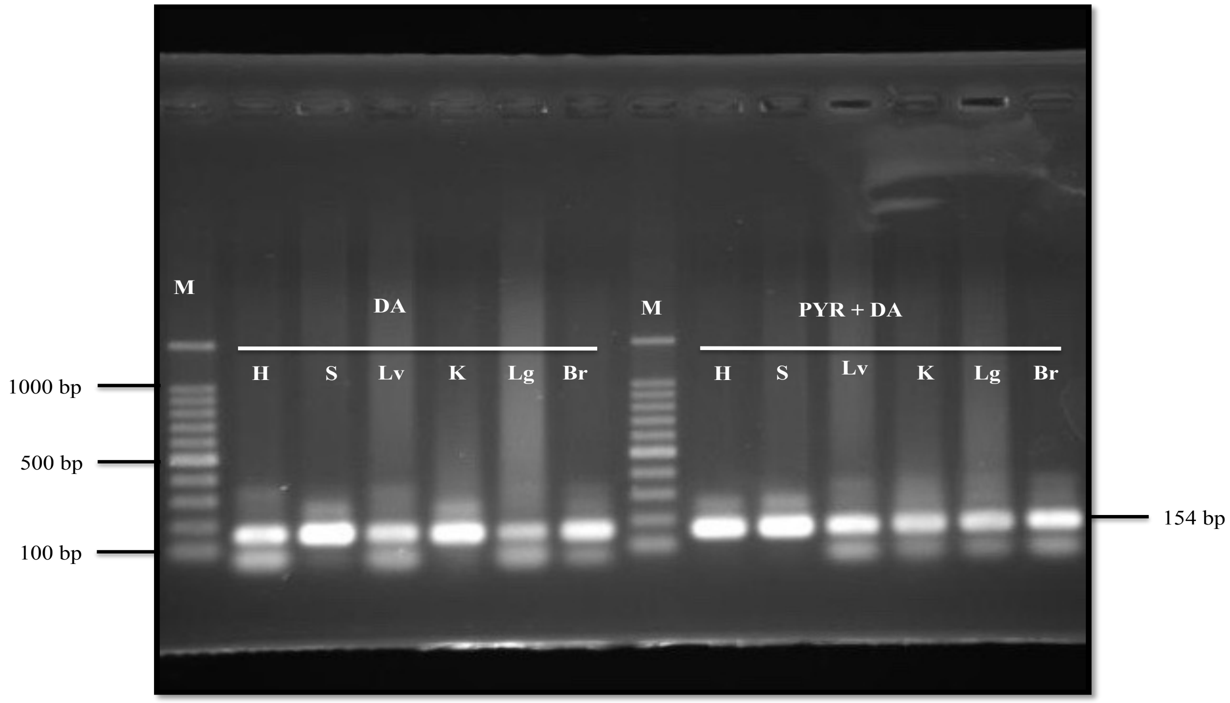

Supplementary Figure S1. Full-length gels for PCR of the ss-rRNA gene in different organs of mice on day 30 post-infection. (a) *Babesia microti*- infected mice that received no treatment (positive control) and non-infected mice (negative control). (b) mice infected with *Babesia microti* after treatment with 25 mg kg $^{-1}$ of diminazene aceturate (DA) and a pyronaridine tetraphosphate (PYR) (85 mg kg $^{-1}$)/DA (10 mg kg

$^{-1}$) combination. PC, positive control; NC, negative control; Br, brain; Lg, lung; K, kidney; Lv, liver; S, spleen; H, heart; M indicates a 100-bp DNA ladder. The expected size of the PCR product was 154 bp.
